# The Response of Tomato Fruit Cuticle Membranes Against Heat and Light

**DOI:** 10.3389/fpls.2021.807723

**Published:** 2022-01-07

**Authors:** José J. Benítez, Ana González Moreno, Susana Guzmán-Puyol, José A. Heredia-Guerrero, Antonio Heredia, Eva Domínguez

**Affiliations:** ^1^Instituto de Ciencia de Materiales de Sevilla, Centro Mixto Consejo Superior de Investigaciones Científicas-Universidad de Sevilla, Seville, Spain; ^2^Departamento de Biología Molecular y Bioquímica, Instituto de Hortofruticultura Subtropical y Mediterránea “La Mayora”, Universidad de Málaga-Consejo Superior de Investigaciones Científicas, Universidad de Málaga, Málaga, Spain; ^3^Departamento de Mejora Genética y Biotecnología, Instituto de Hortofruticultura Subtropical y Mediterránea “La Mayora”, Universidad de Málaga-Consejo Superior de Investigaciones Científicas, Estación Experimental La Mayora, Málaga, Spain

**Keywords:** tomato fruit cuticle membrane, thermal characterization, UV-Vis screening, heat capacity, glass transition, fruit growth and ripening

## Abstract

Two important biophysical properties, the thermal and UV-Vis screening capacity, of isolated tomato fruit cuticle membranes (CM) have been studied by differential scanning calorimetry (DSC) and UV-Vis spectrometry, respectively. A first order melting, corresponding to waxes, and a second order glass transition (*T*_*g*_) thermal events have been observed. The glass transition was less defined and displaced toward higher temperatures along the fruit ripening. In immature and mature green fruits, the CM was always in the viscous and more fluid state but, in ripe fruits, daily and seasonal temperature fluctuations may cause the transition between the glassy and viscous states altering the mass transfer between the epidermal plant cells and the environment. CM dewaxing reduced the *T*_*g*_ value, as derived from the role of waxes as fillers. *T*_*g*_ reduction was more intense after polysaccharide removal due to their highly interwoven distribution within the cutin matrix that restricts the chain mobility. Such effect was amplified by the presence of phenolic compounds in ripe cuticle membranes. The structural rigidity induced by phenolics in tomato CMs was directly reflected in their mechanical elastic modulus. The heat capacity (*Cp*_*rev*_) of cuticle membranes was found to depend on the developmental stage of the fruits and was higher in immature and green stages. The average *Cp*_*rev*_ value was above the one of air, which confers heat regulation capacity to CM. Cuticle membranes screened the UV-B light by 99% irrespectively the developmental stage of the fruit. As intra and epicuticular waxes contributed very little to the UV screening, this protection capacity is attributed to the absorption by cinnamic acid derivatives. However, the blocking capacity toward UV-A is mainly due to the CM thickness increment during growth and to the absorption by flavone chalconaringenin accumulated during ripening. The build-up of phenolic compounds was found to be an efficient mechanism to regulate both the thermal and UV screening properties of cuticle membranes.

## Introduction

The plant cuticle membrane (CM) is a hydrophobic extracellular layer that protects the outermost surface of fruits, leaves, seeds, petals, and green stems from the environment. One of the main functions of CM is to guard the plant against environmental stresses such as harmful irradiation, as well as thermal and mechanical damage and water loss (Domínguez et al., 2011; Heredia-Guerrero et al., 2018). The protective level of the cuticle membrane is conditioned by the amount and assembly of its components, i.e., the polyester cutin matrix, polysaccharides, waxes, and phenolics (López-Casado et al., 2007; Domínguez et al., 2011; Yeats and Rose, 2013). Waxes have been shown to play a major role in the protection against water loss whereas phenolic compounds have proven to be very effective in modulating the mechanical behavior of cuticle membranes (Domínguez et al., 2009; España et al., 2014). Over the last decades, the water permeability and mechanical properties of CMs have been extensively studied (Riederer and Schreiber, 2001; Khanal and Knoche, 2017); however, much less attention has been paid to the analysis of their thermal and UV-Vis screening response and, in particular, to their changes along fruit development.

Though, the structural modifications induced by temperature play a key role in the biophysics of the cuticle membrane, comparatively little work has been done on the thermal characterization of isolated CMs and their components, mostly on tomato fruit (Schreiber and Schönherr, 1990; Luque and Heredia, 1994, 1997; Casado and Heredia, 2001; Matas et al., 2004). Two main thermal events have been reported for isolated CMs, a first order wax melting and second order glass transition. The glass transition (*T*_*g*_) entails a network relaxation and the appearance of conformational changes and segmental mobility within the biopolymer structure. Such rheological alteration of the cuticle membrane is associated with the modification of mass transfer between the epidermal plant cells and the environment (Schreiber and Schönherr, 1990) with foreseeable physiological and ecological consequences. In this sense, the modulation of *T*_*g*_ can be envisaged as an adaptation mechanism of plants to the environment (Matas et al., 2004).

Most of the research on the interaction of UV-Vis radiation with plant CMs has been concentrated on leaves (see reviews by Kolb and Pfündel, 2005; Pfündel et al., 2006; Karabourniotis et al., 2021). However, much less work has been focused on fruits (Ward and Nussinovitch, 1996; Krauss et al., 1997; Kolb et al., 2003; Solovchenko and Merzlyak, 2003). Excessive exposure, particularly to the higher energy UV component, induces photochemical reactions that may cause damage to proteins and the modification of the enzymatic activity that could have a negative effect on quality parameters such as texture, color and organoleptic and nutritional values of produce (Hollósy, 2002; Karabourniotis et al., 2021). However, under moderate UV exposure, the biosynthesis of flavonoids is stimulated (Awad et al., 2000; Luthria et al., 2006) to the point that UV-A irradiation can be proposed as farming and postharvest treatments to improve the antioxidant level of tomato fruits (Luthria et al., 2006; Dyshlyuk et al., 2020). Benefits of moderate UV exposure in the appearance (coloration) and susceptibility to russeting of apple fruit have also been reported (Khanal et al., 2020). Phenolic (either hydroxycinnamic acid derivatives or flavonoids) have been identified as responsible for the UV screening in fruits (Pfündel et al., 2006). In *V. vinifera berries* and in apple fruit, it has been proposed that their accumulation in the epidermal cells controls the UV screening capacity (Moskowitz and Hrazdina, 1981; Solovchenko and Merzlyak, 2003). Meanwhile, the role of cuticular phenolics seems to be restricted to be a primary shield against UV radiation under low levels of solar exposure, but no data on the characterization and response of isolated tomato fruit cuticle membranes to UV-Vis light have been found in the literature.

In this article, we have addressed the study of the thermal and UV-Vis screening properties of isolated tomato CMs and their changes throughout fruit development to provide new insights to the current knowledge in the field.

## Materials and Methods

### Plant Material and Cuticle Membrane Isolation

*Solanum lycopersicum* L. “Cascada” plants were grown from seeds treated with a solution of chlorine bleach 50% (v/v) in distilled water, rinsed and incubated in the dark in a moist environment for several days until germination. Seedlings were then transplanted to plug trays containing 85% coconut fiber substrate and 25% plant-nutrient loaded zeolite and grown in an insect-proof glasshouse. At the four true-leaf growth stage, they were transplanted into soil in a multi-tunnel plastic greenhouse with a 0.5 m within-row and 1.5 m between-row spacing. Plants were watered when necessary using a nutrient solution, supported by string and pruned to a single stem. To harvest fruits at specific time-points, flowers were labeled at anthesis and vibrated to ensure fruit set.

Cuticle membranes were enzymatically isolated from tomato fruits at different stages of development following an enzymatic protocol (Petracek and Bukovac, 1995). Tomatoes were halved and immersed in an aqueous solution of a mixture of fungal cellulase (0.2% w/v) and pectinase (2.0% w/v) in sodium citrate buffer (50 mM, pH 3.7) and incubated for a week at 35°C with continuous agitation. Sodium azide (1 mM) was added to the citrate buffer to prevent microbial growth. After this period, flesh tissue could easily be removed. The cuticle membranes, with remnants of internal tissue, were incubated for another week in the enzymatic solution. At this point, the CM apparently did not have any remaining internal tissue debris. Another change in enzymatic solution was carried out then, and the samples incubated for a 3rd week. Cuticle membranes were then visually inspected and, if any residual tissue was still attached, incubated again with fresh enzymatic solution. This procedure was employed to avoid peeling the fruits or any other mechanical practice that could compromise CM’s integrity, especially in immature fruits. Samples were then rinsed with distilled water, air dried on a flat Teflon surface, moved to Petri dishes and stored under dry conditions (Domínguez et al., 2008; España et al., 2014). Waxes were removed by treatment with a hot (∼60°C) mixture of chloroform: methanol (2: 1, v:v) for 2 h. Polysaccharides were eliminated from previously dewaxed cuticle membranes to yield the cutin fraction by refluxing them in a 6 M HCl aqueous solution for 12 h (Matas et al., 2004). The developmental stage of fruits is defined as days after anthesis (daa). Samples were characterized at the hydration degree corresponding to their stabilization at room conditions (typically 20°C and 45% RH).

### Infrared Spectroscopy

Attenuated Total Reflection (ATR-FTIR) spectra were obtained using a single reflection ATR accessory (MIRacle ATR, PIKE Technologies, Madison, WI, United States) with a diamond crystal at 45° incidence and coupled to a FT-IR spectrometer (FT/IR-6200, Jasco, Tokyo, Japan). All spectra were acquired with a liquid nitrogen cooled MCT detector in the 4000–6000 cm^–1^ range at 4 cm^–1^ resolution and by accumulating 50 scans. Band area calculation and ATR penetration depth correction were performed using the Jasco SpectraManager software V.2 (Jasco Corporation, Tokyo, Japan). Three samples at each daa stage were analyzed.

### Mechanical Characterization

Uniaxial tensile tests were carried out with a Criterion 42 (MTS Systems, Eden Prairie, MN, United States) machine equipped with a low force (10 N) cell. Rectangular uniform pieces (5 mm × 15 mm) were cut and elongated at room conditions using a crosshead speed of 0.2 mm/min with a clamping distance of 7 mm. The Young’s modulus (E) was calculated from the maximum slope of the stress-strain curve (typically around 1–2% strain). Ten replicates per specimen were analyzed.

Storage moduli (E′) were obtained at room conditions by Dynamic Mechanical Analysis (DMA) measurements using a Q800 analyzer (TA Instruments, New Castle, DE, United States) in tension mode. In these tests, the sample is stressed with a low amplitude sinusoidal force and the strain response is simultaneously decomposed into an instantaneous in-phase (elastic) and a delayed out-of-phase (viscous) components. The storage modulus (E′) corresponds to the pure elastic response of the sample. Experiments were repeated with five samples at each daa stage. A more detailed description of the procedure is provided elsewhere (Benítez et al., 2021).

### Thermal Characterization

Samples for Differential Scanning Calorimetry (DSC) measurements were prepared by stacking punched 4.5 mm Ø discs inside a TZero (TA Instruments, New Castle, DE, United States) aluminum pan covered with a non-hermetic lid. The final sample weight was about 5 mg and was accurately determined for each specimen with 0.01 mg precision. The DSC equipment was a Q-20 (TA Instruments, New Castle, DE, United States) previously calibrated with indium (for temperature and enthalpy) and sapphire (for heat capacity) references. DSC thermograms consisted in a heat-cool-heat cycle from −70 to 100°C at 10°C/min under N_2_ flow at 50 mL/min. Glass transition temperature (*T*_*g*_) values were calculated from the second heating by the inflection method using the in-built TA Instruments Universal Analysis 2000 v. 4.5A software (TA Instruments-Waters LLC). Values were averaged from three measurements. The absence of temperature effects on the *T*_*g*_ was confirmed by the steadiness of values after sample pretreatments up to 150°C.

The heat capacity was obtained by non-isothermal modulated DSC (M-DSC) in the −5 to 55°C range after the DSC heat-cool-heat cycle. In M-DSC, a sine wave temperature signal of ± 1°C amplitude and 120 s period was superimposed to a linear temperature ramp of 1°C/min and the enthalpy variation was recorded. The reversible specific (*Cp*_*rev*_) was calculated at each temperature by dividing the amplitude of the enthalpy variation signal by the sample weight. To minimize the contribution from the aluminum pan, those used for the sample and the reference were selected to differ in less than 0.05 mg. The sample consisted in circular pieces from about 20–25 different fruits, which is considered to be statistically representative, and no measurement repetition was performed.

### UV-Vis Spectrometry

UV-Vis spectra were recorded using a Cary 300 (Agilent Technologies, Santa Clara, CA, United States) spectrophotometer equipped with a DRA-CA-30I integrating sphere (Labsphere Inc., North Sutton, NH, United States) and using a 8° wedge to collect the total (diffuse and specular) reflectance. Cuticle membranes were mounted on aluminum mask holders coated with barium sulfate (ODP97, Gigahertz-Optik GmbH, Türkenfeld, Germany) and always irradiated on the outer side, thus resembling the orientation of the fruit with respect to sunlight in nature and to account for the effect of the epicuticular wax layer on the intensity of reflected light. The total transmitted and reflected light intensity were collected in the 200–800 nm range. The absorbance spectrum (A) was obtained according to equation (I) (Solovchenko and Merzlyak, 2003):


(I) A=log((I-OI)R/I)T

where (I_*O*_) is the incident intensity (defined as 100%) and (I_*R*_) and (I_*T*_) the percentages of reflected and transmitted light at each wavelength, respectively.

Reflectance in a given region is defined as:


(II) R=100-RTavg

where RT_avg_ is the average of total reflectance values (as percentage) in that region. Analogously, the blockage is calculated by:


(III) Blockage=100-TTavg

where TT_avg_ is the average of total transmittance values (in percentage) in the defined region.

### Statistical Analysis

Data are expressed as mean ± 1 SE and significance was determined by one-way ANOVA. Pearson’s correlation analysis was performed using OriginPro, Version 2019 software (OriginLab Corporation, Northampton, MA, United States). Letters indicate significant differences (*P* < 0.05).

## Results

### Fruit Development and Physical and Chemical Modification of the Cuticle Membrane

**FIGURE 1:**
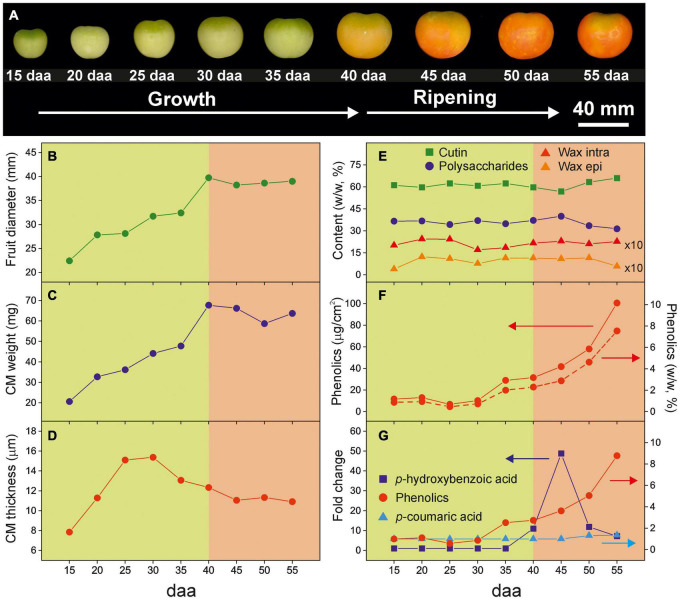
**(A)** Visual aspect and parameters modification along tomato fruit growth and ripening [**(B)** fruit diameter, **(C)** CM weight, **(D)** CM average thickness, **(E)** CM composition, **(F)** phenolics content and **(G)** relative evolution of hydroxycinnamic acids and total phenolic compounds in the cuticle membrane] (adapted from Domínguez et al., 2008 and España et al., 2014).

Figure 1 summarizes some of the physical, morphological and chemical modifications in tomato Cascada fruits and their isolated CMs along development. From 15 to 40 daa the fruit experiments a noticeable increment of size (Figures 1A,B). Such growth stage is characterized by a predominant green color tone, an increment of CM weight (Figure 1C) and thickness (Figure 1D) and relatively low phenolics content (Figure 1F). Above 40 daa, the fruit size remains constant and the average cuticle membrane thickness decreases slightly. However, this ripening period is characterized by an intense change in color and the accumulation of phenolic compounds in the CM. The overall cuticle membrane composition along the fruit development remains almost unaltered with cutin as the most abundant fraction followed by polysaccharides. Waxes and phenolics are minor components (Figures 1E,F). However, in the ripening stage, a small reduction in polysaccharides can be appreciated.

### ATR-FTIR Analysis of Isolated Cuticle Membrane and Cutins

**FIGURE 2:**
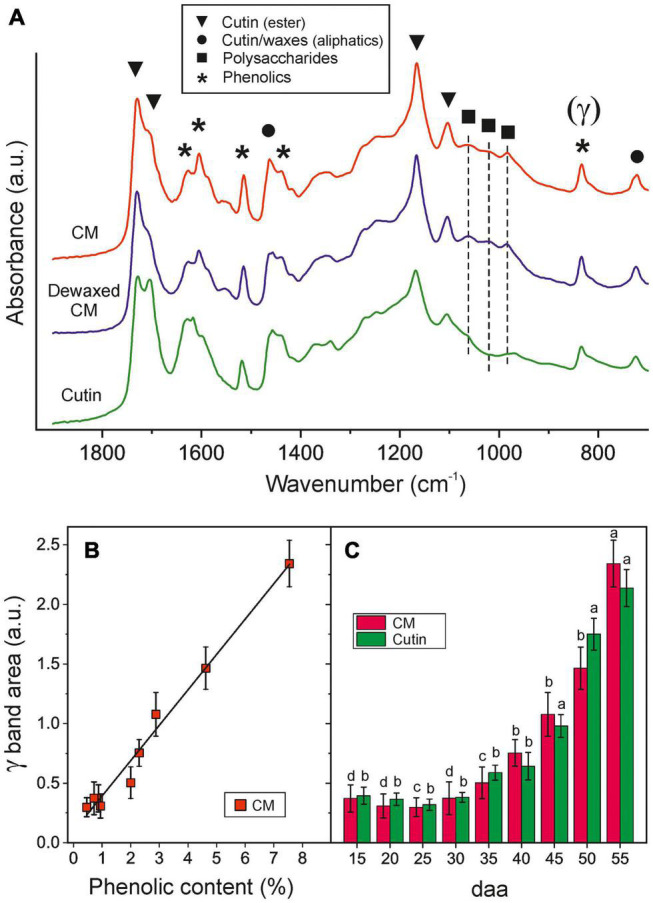
**(A)** ATR-FTIR spectra of isolated and dewaxed CMs and cutins of ripe tomato fruit. Characteristic bands of cutin, polysaccharides, waxes and phenolics are labeled with symbols. **(B)** Correlation between the phenolics content and the ATR-FTIR γ band area in cuticle membranes. **(C)** Evolution of phenolics content by means of the ATR-FTIR γ band area in tomato CMs and cutins along fruit growth and ripening. Error bars represent ± 1 SE and letters indicate significant differences (*P* < 0.05).

Tomato fruit CMs throughout development contain a ∼55–62% w/w of cutin (a fatty polyester matrix) with a 0.4–1.2% w/w and ∼2–2.4% w/w of epicuticular and embedded waxes, respectively, and a variable amount of phenolic compounds (0.3–7% w/w) that strongly increases with ripening (Figures 1E,F). They also have an important remnant polysaccharide fraction from the cell walls (31–40% w/w). The presence of such fractions can be detected in the ATR-FTIR spectra. Figure 2A shows the 700–1900 cm^–1^ region where characteristic ester bands ν(C=O) at 1731 and 1715 cm^–1^ (hydrogen bonded) as well as ν(C-O-C) at 1168 and 1105 cm^–1^ are observed (Heredia-Guerrero et al., 2014). Aliphatic chains in cutin and waxes are characterized by intense peaks at 2926 and 2854 cm^–1^ [ν_*a*_(CH_2_) and ν_*s*_(CH_2_), respectively, not shown] and weaker (CH_2_) deflection modes at 1463 (scissoring) and 725 cm^–1^ (rocking). The presence of polysaccharides is revealed by ν(C-O), ν(C-C) and δ(C-O) peaks at 1053, 1034 cm^–1^ and 984 cm^–1^, corresponding to cellulose, hemicellulose and pectin, respectively (Szymanska-Chargot and Zdunek, 2013). Phenolic compounds are detected by their ν(C=C) (1627 cm^–1^), ν(C-C) (1605, 1515 and 1436 cm^–1^) and γ(C-H) (835 cm^–1^) modes (Ramírez et al., 1992; España et al., 2014).

Cuticle membranes dewaxing has no detectable effect on the ATR-FTIR spectrum, which is consistent with the low wax content. On the other side, polysaccharide removal is evident from the intense reduction of bands at 1053 and 984 cm^–1^ and the elimination of the 1034 cm^–1^ peak in cutins. Bands corresponding to phenolics remain unaltered, which suggests that embedded phenolics withstand both the organic solvent extraction and the polysaccharide hydrolysis. The normalized γ band area at 835 cm^–1^ has proven to be an accurate parameter to quantify the amount of phenolics *in situ* with no need for CM processing and depolymerization (Figure 2B). Accordingly, ATR-FTIR data confirmed the intense accumulation of phenolics in the ripening stage (40–55 daa) and their affinity for the cutin matrix (Figure 2C).

### Differential Scanning Calorimetry of Tomato Cuticle Membranes and Cutins

**FIGURE 3:**
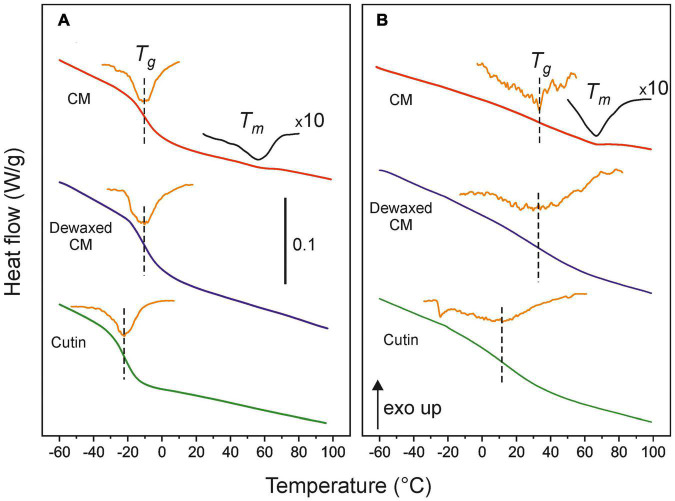
DSC thermograms of isolated and dewaxed cuticle membranes and cutins of **(A)** immature green (20 daa) and **(B)** red ripe (55 daa) tomato fruits. The orange line is the derivative of the heat flow signal and marks the *T*_*g*_ value. For CMs, an expanded heat flow trace (×10) is included to facilitate the visualization of the wax melting peaks. The bar in **(A)** represents a 0.1 W/g heat flow variation.

Figure 3 shows the DSC thermograms of isolated CMs as well as those after dewaxing and polysaccharide removal (cutin) for immature green (20 daa) (Figure 3A) and red ripe (55 daa) (Figure 3B) tomato fruits. The full thermogram series are compiled in Supplementary Figure 1. As observed, the main thermal event is the second-order transition from a rigid glassy state to a more relaxed and dynamic rubbery conformation (*T*_*g*_). Such glass transition is characteristic for amorphous polymers and displays a sharp profile in immature and mature green CMs. The glass transition becomes systematically broader, less defined and displaced toward higher temperature while ripening. Dewaxing has virtually no effect on *T*_*g*_ values of CMs, however, the removal of polysaccharides significantly diminishes the *T*_*g*_ of the cutin fraction. In addition to the glass transition, in isolated cuticle membranes, a weak endothermic peak around 55°C caused by the melting of the small fraction of intra and epicuticular waxes (∼3% w/w) can be detected. As expected, this melting is absent in dewaxed CMs and in cutins.

**FIGURE 4:**
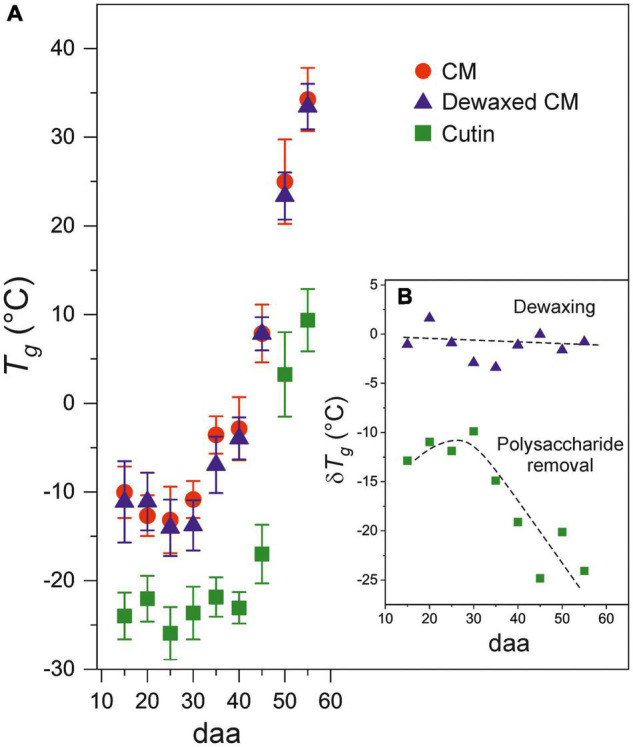
**(A)** Evolution of *T*_*g*_ for CMs, dewaxed CMs and cutins along fruit development. **(B)** *T*_*g*_ variation (δ*T*_*g*_) after dewaxing (triangles) and polysaccharide removal (squares) from cuticle membranes. Error bars represent ± 1 SE.

The developmental time course of *T*_*g*_ values of CMs, dewaxed CMs and cutins is observed in Figure 4A. The plots show the systematic lower values of cutins with respect to CMs and reveal the sharp increment of the glass transition temperature above 35 daa and up to full ripening at 55 daa. The inset (Figure 4B), remarks the reduction of *T*_*g*_ (δ *T*_*g*_) after wax and polysaccharide removal. Such reduction is variable and very low after dewaxing, but very noticeable after polysaccharide removal, particularly during ripening (above 35 daa).

**FIGURE 5:**
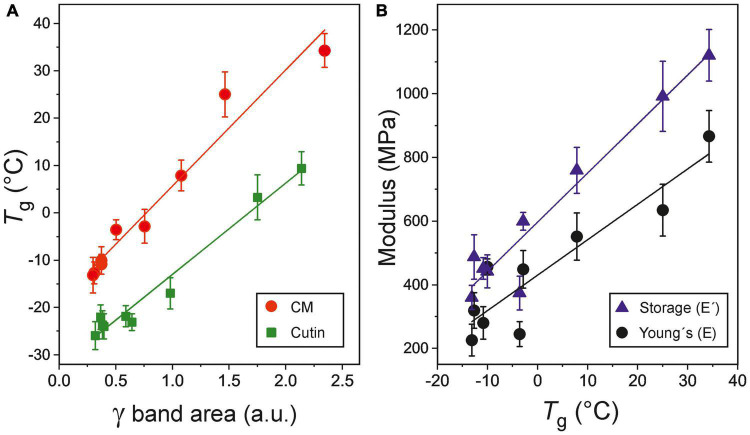
**(A)** Correlation between the glass transition temperature (*T*_*g*_) and phenolics content, as monitored by the ATR-FTIR γ band, in CMs and cutins of tomato fruit. **(B)** Direct relationship between the Young’s (E) and storage (E′) moduli and the glass transition temperature (*T*_*g*_) in tomato fruit cuticle membranes. Error bars represent ±1 SE.

A significant and positive correlation between the areas of the ATR-FTIR γ band, an indicator of phenolic content, and *T*_*g*_ values can be observed for isolated CMs and cutins (*r* = 0.978, *P* < 0.01 and *r* = 0.985, *P* < 0.01, respectively) along fruit development (Figure 5A). Such structural rigidity induced by phenolics in tomato cuticle membranes is also reflected in their mechanical response with a positive and significant correlation between the *T*_*g*_ and the **Young’s** (E) and storage (E′) moduli (*r* = 0.916, *P* < 0.01 and *r* = 0.966, *P* < 0.01, respectively) (Figure 5B).

### Reversible Heat Capacity of Tomato Fruit Cuticle Membranes and Cutins

**FIGURE 6:**
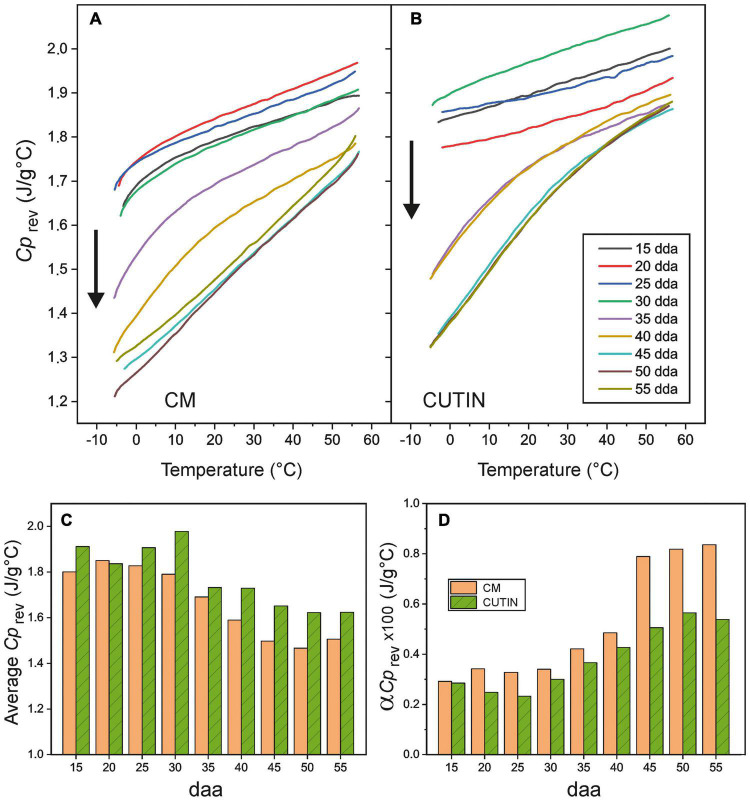
Reversible heat capacity values (*Cp*_*rev*_) for **(A)** cuticle membranes and **(B)** cutins of tomato fruit in the (–5 to 55°C) temperature interval. Arrows highlight the curve evolution with daa time. **(C)** Average heat capacity values in the (0–50°C) interval and **(D)** slope of the linear region of the curves (α*Cp*_*rev*_) for CMs and cutins along fruit growth and ripening.

Reversible heat capacity (*Cp*_*rev*_) values versus temperature for isolated tomato CMs and cutins are shown in Figures 6A,B, respectively. As observed, the heat capacity grows almost linearly with temperature. The main deviation from this trend is caused by the occurrence of the glass transition within the temperature range analyzed. Deviations are particularly visible in CMs because their *T*_*g*_ values are higher than those of the corresponding cutins (Figure 4); also in samples where the transition in very sharp, as in 35 and 40 daa CMs and in cutins above 35 daa (see Supplementary Figure 1). Additionally, in cuticle membranes and above 50–55°C, the beginning of wax melting causes an increment of heat capacity.

In general, the *Cp*_*rev*_ curves displace toward lower values and increment their slope with fruit development. Such effects can be better visualized in Figures 6C,D, where the average *Cp*_*rev*_ value in the (0 to 50°C) interval and the slope of the curve (α*Cp*_*rev*_) above the glass transition are, respectively, plotted versus fruit daa. The (0 to 50°C) interval for average *Cp*_*rev*_ calculation has been selected as representative for the temperature range experimented by fruits along growth and ripening.

It is interesting to notice that the average *Cp*_*rev*_ of CMs are lower than those of cutins, Figure 6C. This is very likely due to the contribution of the polysaccharide fraction of the cuticle membrane, as the heat capacities of cellulose, hemicellulose and pectin are reported to be in the 1.2–1.5 J/g°C range at room temperature (Blokhin et al., 2011; Domínguez et al., 2011; Mohd Rasidek et al., 2018; Qi et al., 2020). Considering an average *Cp*_*rev*_ = 1.75 J/g°C for cutin and 1.3 J/g°C for polysaccharides and their relative abundances in CMs (64 and 36%, respectively), the calculated *Cp*_*rev*_ for cuticle membranes is 1.59 J/g°C. Thus, polysaccharides cause a theoretical reduction of 0.16 J/g°C in the cutin matrix, quite close to the average 0.12 J/g°C experimental difference observed (Figure 6C).

It is also worth noticing that average *Cp*_*rev*_ values slightly decrease in the ripening stage. This is partially due to the fact that heat capacity increases noticeably when crossing the glass transition. CMs of immature and mature green fruits in the 0–55°C range are always above their *T*_*g*_, and, consequently, the contribution of higher *Cp*_*rev*_ values is larger than in cuticle membranes of ripe fruits.

### UV-Vis Absorption and Blocking Capacity of Tomato Fruit Cuticle Membranes

**FIGURE 7:**
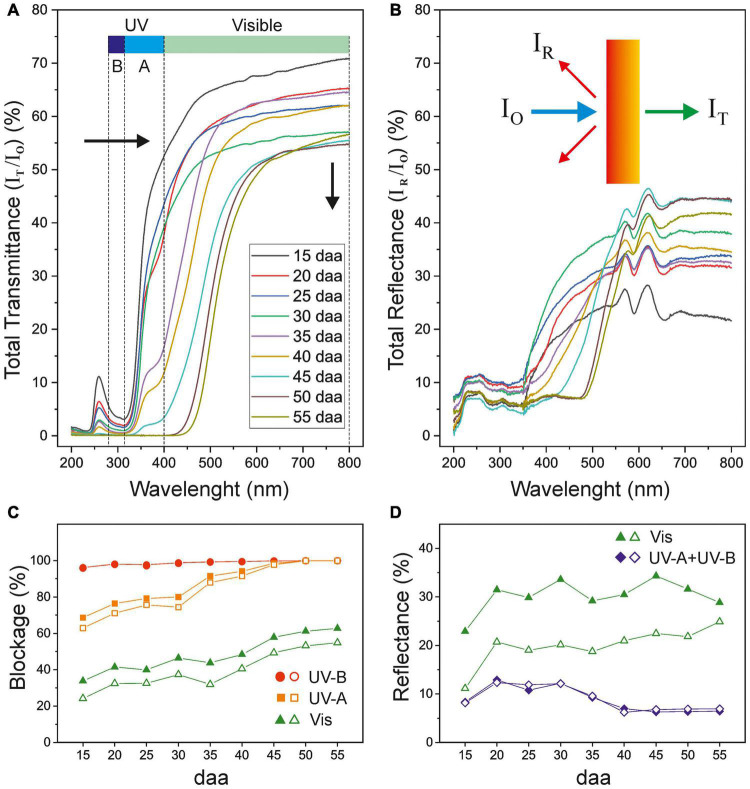
**(A)** Total transmittance and **(B)** total reflectance spectra of isolated tomato CMs along fruit growth and ripening. Black arrows in **(A)** show the spectra displacement along fruit development. **(C)** Blockage and **(D)** reflectance percentages of UV and visible light of tomato fruit cuticle membranes during growth and ripening. Open symbols correspond to dewaxed CMs.

Figure 7A shows the changes in the total transmittance UV-Vis spectra of tomato CMs during growth and ripening. The shape of the curves resembles the pattern of a selective optical filter with a sharp transition from a low transmission region in the UV to a highly transparent one in the visible range (Krauss et al., 1997; Karabourniotis et al., 2021). The cut-off wavelength progressively displaces during fruit development from about 350 nm for the immature and mature green CMs to 525 m for red ripe ones. The maximum transmittance gradually decreases during the whole 15–55 daa period from about 70 to 53%. Interestingly, a small peak of transmittance was detected around 260 nm, within the UV-C region, that slowly decreased with development.

The UV-Vis blockage capacity of tomato cuticle membranes displays a complex trend with fruit growth and ripening (Figure 7C). While the screening against UV-B is extremely high with an average value of 99% for the whole developmental period, a similar level of protection against UV-A is only attained in CMs of breaker and ripe tomatoes (45–55 daa). Although CMs of immature and mature green fruits (15–30 daa) show an average ∼75% UV-A blockage, with a small increase trend, a steep transition from very low to high transmittance was observed for these cuticle membranes within the UV-A region (Figure 7A). A similar small increase in the Vis blocking is detected within the 15–30 daa period, with a plateau at 35 daa, and a more prominent increase during ripening. CM dewaxing did not have an effect on UV-B blocking at any stage of development and a very minor influence in UV-A blockage. A higher, yet still slight, effect is observed for Vis screening indicating that waxes play a small role in blocking the incident radiation.

The outer surface of tomato CMs shows little reflectance within the UV-B and most of the UV-A regions while it is moderately reflective in the visible range (400–800 nm). Maximum values of total reflectance (RT) ranges from 20 to 45% but without any clear relation with the developmental stage (Figure 7B). Reflectance is conditioned by many factors such as the CM surface topography, the total wax content and the structure and composition of the epicuticular wax layer (Vogelmann, 1993; Ward and Nussinovitch, 1996; Petit et al., 2014). A more detailed analysis of the reflectance of tomato CMs as a function of fruit growth and ripening is provided in Figure 7D, where values within each spectral region have been averaged. As observed, average reflectance values in the visible region are quite constant (∼30%). However, dewaxing causes an important 35% reduction (values ∼20%), which confirms the important contribution of waxes to the ability of cuticle membranes to reflect visible light. On the other hand, UV reflectance (UV-A + UV-B) is significantly low (∼9%) and wax removal does not have any effect, as reported by Koch and Barthlott (2006).

**FIGURE 8:**
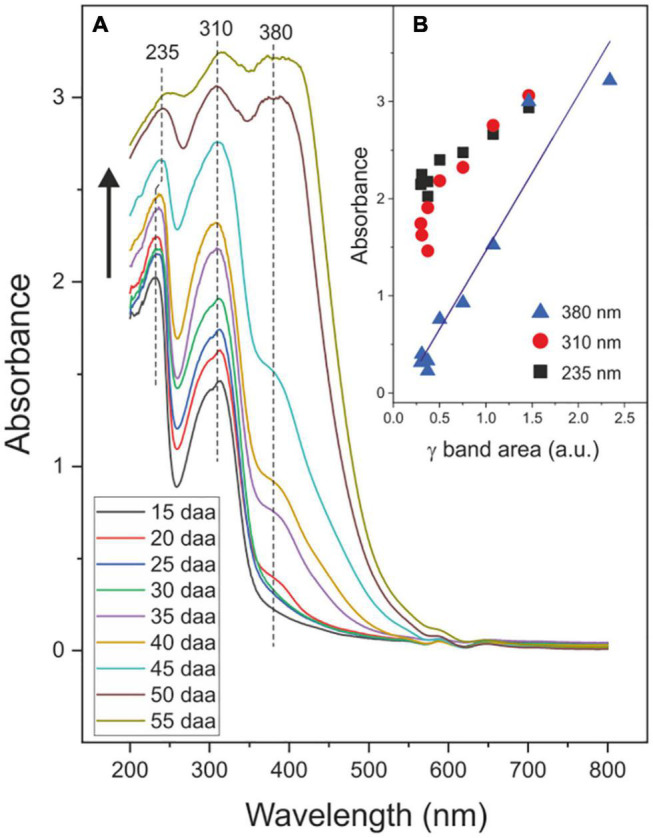
**(A)** Absorbance spectra of isolated tomato CMs during fruit growth and ripening. Black arrow indicates the evolution of spectra with the developmental stage. **(B)** Relationship between the absorbance at 235, 310, and 380 nm and the phenolic content *via* the quantification of the ATR-FTIR γ band.

The absorbance spectra of tomato CMs during fruit development are shown in Figure 8A. Peaks detected at 235, 310, and 380 nm are assigned to the π→π*electron transitions in conjugated (C = C) bonds, hydroxycinnamic acid derivatives and flavonoids, respectively (Cockell and Knowland, 1999; Kolb et al., 2003; Pfündel et al., 2006). The absorbance at 380 nm increases with fruit development and significantly and positively correlates (*r* = 0.958, *P* < 0.01) with phenolics accumulation (Figure 8B). Absorbance at 235 and 310 nm seems to follow an analogous trend though the accuracy of values may be conditioned by difficulties in assessing the base line of spectra and the proximity to band saturation.

## Discussion

### Phenolic Compounds and Polysaccharides Modulate the Glass Transition Temperature of Tomato Fruit Cuticle Membranes

Differential scanning calorimetry results demonstrate that the glass transition temperature (*T*_*g*_) of tomato CMs clearly depends on the developmental stage of the fruit. Thus, in immature and mature green fruits, the *T*_*g*_ occurs at temperatures below zero indicating that, during the growing period the cuticle membranes are in a more viscous and fluid state. However, during ripening, the *T*_*g*_ strongly increases to temperatures that reach 20–35°C, well within the average temperature of tomato plant growth. This implies that during daily (and seasonal) fluctuations in temperature, the fruit CM of ripening tomatoes can be oscillating between a rigid and a viscous state. It is well known that water acts as a plasticizer reducing the *T*_*g*_ of a sample. Although the analysis has been carried out with dry samples and, in the fruit, the CM is expected to have a certain degree of hydration, the magnitude of the reported *T*_*g*_ reduction with hydration (Matas et al., 2004) is not expected to alter this conclusion.

Intracuticular waxes have been reported to behave as fillers within the cuticule membrane framework (Luque and Heredia, 1994; Petracek and Bukovac, 1995; Tsubaki et al., 2013). Fillers occupy the space in between chains in the CM structure and reduce the free volume available for motions. Consequently, intracuticular waxes can shift the transition from the glassy to the viscous state to higher temperatures. This is in agreement with the reduction of ∼1.5°C in the *T*_*g*_ observed in dewaxed CMs at all stages of development. This is a small reduction in *T*_*g*_ but it should be taken into account that waxes are minor CM components in tomato (Domínguez et al., 2008). Thus, in species with a higher intracuticular wax load, a more prominent change in *T*_*g*_ could be expected.

Phenolic compounds have also been described as fillers that could explain the increment of *T*_*g*_ in CMs (Luque and Heredia, 1994). Whereas the amount of waxes does not exhibit significant changes during fruit development (Figure 1E), this is not the case for phenolic compounds (Figure 1F). Tomato fruit cuticle membranes display a low amount of phenolics during most of the growing period that notably increases during ripening reaching a maximum at the red ripe stage. Thus, phenolics would have a minor contribution to the *T*_*g*_ during growth, but play a major role in the dramatic increase of *T*_*g*_ during ripening, as indicated by the linear correlation in Figure 5A. However, the role of phenolics does not seem to be merely restricted to fill nanomeric cavities within the cuticle membrane by bonding to free groups in the polymeric framework. Tentatively, they are also involved in bridging polymeric chains as cross-linkers and thus incrementing the elastic modulus (Domínguez et al., 2009; España et al., 2014; Benítez et al., 2021). Such behavior would explain the observed correlation between the glass transition temperature and mechanical elasticity (Figure 5B). It is worth mentioning that “rigidity” in terms of restriction to the internal and segmental motions of chains induced by temperature (*T*_*g*_) is not the same concept as “rigidity” as the structure response to an external mechanical stress (elastic modulus). However, both parameters generally correlate in cross-linked polymers.

The polysaccharide fraction of the cuticle membrane seems to have an important effect on the glass transition since its removal causes a notable decrease in *T*_*g*_. Thus, during the growing period, when the amount of phenolics is low, the extraction of polysaccharides reduces the *T*_*g*_ value about 11°C (Figure 4B). This is compatible with a highly interwoven distribution of polysaccharides within the polyester cutin framework in the CM, giving rise to a closer arrangement of chains that restricts their internal motions. Furthermore, the accumulation of phenolics during ripening notably and progressively increment the *T*_*g*_ reduction after polysaccharide removal (Figure 4B), which suggests an enhanced bonding between polysaccharides and cutin phases mediated by phenolic compounds. An analogous model has been proposed to explain the mechanical reinforcement induced by phenolics in tomato fruit cuticle membranes (Benítez et al., 2021). The analysis of the FT-IR data of cutin samples can provide additional support to the hypothesis of a strong binding or interaction between polysaccharides and the cutin polyester matrix. Thus, the ATR-FTIR spectrum of cutin shows the development of a band at 1704 cm^–1^ in parallel to the removal of cellulose, hemicellulose and pectin from the CM (Figure 2A). This band can be assigned to new free carboxylic acid groups resulting from the disassembling of polysaccharides. Moreover, ATR-FTIR analysis did not show any detectable degradation of the polyester matrix or loss of the phenolic peaks, an indication that CM dewaxing and polysaccharide removal by hydrolysis are selective and effective treatments. Consequently, the accumulation of phenolics, and their interaction with both the cutin and the polysaccharide fractions, could be a strategy to regulate the transition of cuticle membranes from a rigid to a viscous state and to adapt their rheology to the requirements of fruit development. Therefore, during fruit growth, the CM is always in the viscous state, which facilitates molecular diffusion processes. As the fruit ripens, the transition temperature threshold notably increases and the mass transfer across the CM would be reduced and become highly dependent on the environmental temperature and its daily fluctuations.

### The Cuticle Membrane Helps to Regulate the Heat Transference Between the Fruit and the Environment

The heat capacity of tomato CMs within the 5–50°C range is significantly higher than the one of atmospheric air (∼1 J/g°C) (Figure 6). This means that under a thermal flux from the external environment, the cuticle membrane is capable to act as a heat sink and to moderate the temperature increment of the internal plant tissues. Though the amount of CM is small if compared with the total mass of the fruit, the thermal regulatory capacity of cuticle membranes may contribute to palliate potential temperature damages to the plant. Interestingly, the average heat capacity of cutins and CMs is reduced during ripening and coinciding with phenolic accumulation (Figure 6C). However, such decrease can be compensated with the higher *Cp*_*rev*_ increment with temperature (α*Cp*_*rev*_), particularly in cuticle membranes (Figure 6D). This means that in hot environments, the thermal regulation of ripe fruit CMs reaches similar values to those of immature and mature green ones.

### Tomato Fruit Cuticle Membranes Effectively Block UV Radiation by Absorption

The tomato fruit CM prevents most of the harmful UV-B radiation to reach the cell and provides a very efficient barrier that virtually blocks UV-B light. This is in good agreement with the reported literature on the cuticle membrane of woody species (Krauss et al., 1997). Interestingly, this almost complete protection is present from the very early stages of growth. However, the blockage of UV-A is clearly dependent on the stage of development, with CMs from immature green fruits displaying the highest transmittance and a virtually zero transmittance at the red ripe stage. Considering the low reflectance of tomato CMs in the UV region (Figure 7D), the blockage capacity in such region has to be ascribed to the absorption by phenolics. However, whereas in the UV-B region the small amount of phenolics present in tomato fruit CMs from early stages of development (España et al., 2014) may be enough for a total reduction of transmittance, this is not the case for the UV-A region of the spectra. Indeed, an increase in UV-A blockage is observed within the 15–30 daa period of development, although the amount of CM phenolics remains relatively constant (España et al., 2014). A more detailed analysis reveals a significant and positive correlation (*r* = 0.965, *P* < 0.05) between the UV-A blockage and the average thickness of the cuticle membrane in the 15–30 daa period (Supplementary Table 1 and Supplementary Figure 2). Hence, it can be concluded that, at low phenolics content, the modification of the UV-A blockage capacity mainly depends on CM thickness. However, such UV-A blockage-thickness relationship is not reproduced for CMs during ripening since thickness does not increase and yet the blockage is almost 100%. It should be reminded that tomato fruit CMs accumulate the flavonoid chalconaringenin during ripening (España et al., 2014). Whereas cinnamic acids absorb within the UV-B region, flavonoids have been reported to mainly absorb within the UV-A region (Kolb et al., 2003; Pfündel et al., 2006). Therefore, the complete blockage of UV-A observed in CMs from ripening tomato fruits could be attributed to the chalconaringenin accumulated as the tomato changes from mature green to red ripe, and not to the additional increase of cinnamic acid derivatives also observed during ripening (Figure 1G). Phenolic compounds have been reported in the epi and intracuticular wax fractions and suggested to play an important role in the optical properties of the cuticle membrane (Karabourniotis and Liakopoulos, 2005). However, it can be concluded that waxes (both intra and epicuticular) contributes very little to the UV screening capacity of tomato fruit CMs, an almost negligible 0.2% in the UV-B region and around a 5% in UV-A at the immature and mature green stages that diminishes as the fruit ripens.

In summary, the tomato fruit cuticle membrane seems to protect the epidermal cells from harmful UV light by a combined strategy of phenolic accumulation and CM thickness modification; with cinnamic acid derivatives protecting from UV-B and CM thickness and chalconaringenin accumulation playing a role in UV-A blockage during fruit growth and ripening, respectively. Therefore, the accumulation of phenolics in the cuticle membrane is a very efficient tool to modulate the amount of UV radiation that reaches the internal tissues and constitutes a strategy of plants to adapt to environmental sunlight levels.

## Data Availability Statement

The raw data supporting the conclusions of this article will be made available by the authors, without undue reservation.

## Author Contributions

JB, ED, and AH conceived and planned the research. JB and AGM performed the experiments. SG-P helped in the analysis of UV-Vis data. JB, JH-G, and ED wrote the article with contributions and supervision of all authors. All authors approved the submitted text.

## Conflict of Interest

The authors declare that the research was conducted in the absence of any commercial or financial relationships that could be construed as a potential conflict of interest.

## Publisher’s Note

All claims expressed in this article are solely those of the authors and do not necessarily represent those of their affiliated organizations, or those of the publisher, the editors and the reviewers. Any product that may be evaluated in this article, or claim that may be made by its manufacturer, is not guaranteed or endorsed by the publisher.
