# Supplementary material for: The Response of Tomato Fruit Cuticle Membranes Against Heat and Light
Source: Front Plant Sci. 2022 Jan 7;12:807723. doi: 10.3389/fpls.2021.807723 (PMC8777011; doi:10.3389/fpls.2021.807723)

**SUPPLEMENTARY INFORMATION**

**The Response of Tomato Fruit Cuticles against Heat and Light**

***José J. Benítez^1*^, Ana González Moreno^2^, Susana Guzmán-Puyol^3^, José A. Heredia-Guerrero^3^, Antonio Heredia^2^ and Eva Domínguez^3^***

*^1^ Instituto de Ciencia de Materiales de Sevilla, Centro Mixto Consejo Superior de Investigaciones Científicas-Universidad de Sevilla, Seville, Spain.*

*^2^ Instituto de Hortofruticultura Subtropical y Mediterránea “La Mayora”, Universidad de Málaga-Consejo Superior de Investigaciones Científicas, Departamento de Biología Molecular y Bioquímica, Universidad de Málaga, Málaga, Spain*

*^3^ Instituto de Hortofruticultura Subtropical y Mediterránea “La Mayora”, Universidad de Málaga-Consejo Superior de Investigaciones Científicas, Departamento de Mejora Genética y Biotecnología, Estación Experimental La Mayora, Málaga, Spain*

**Supplementary Figure S1.** DSC thermogram evolution along fruit growth and ripening of isolated, dewaxed and polysaccharide extracted (cutins) tomato cuticles.


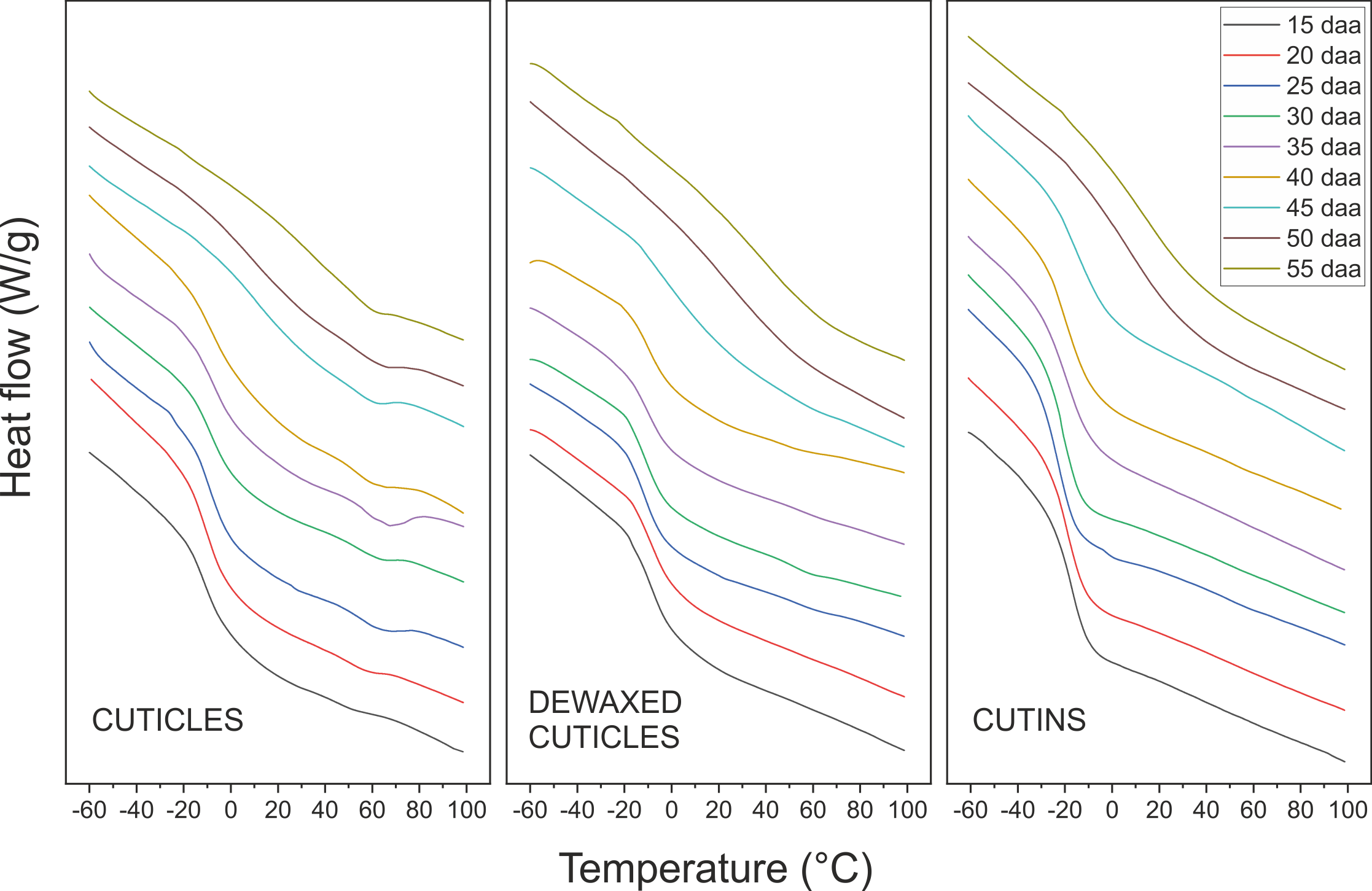


**Supplementary Table 1.** Weight per area (w), density (d) and average thickness (t) values for isolated tomato cuticles along fruit growth and ripening (daa). (w) and (d) are extracted from Domínguez, E., López-Casado, G., Cuartero, J. and Heredia, A. (2008). Development of fruit cuticle in cherry tomato (*Solanum lycopersicum*). *Funct. Plant Biol.* 35, 403-411. doi: 10.1071/FP08018 and España, L., Heredia-Guerrero, J. A., Segado, P., Benítez, J. J., Heredia, A. and Domínguez, E. (2014). Biomechanical properties of the tomato (*Solanum lycopersicum*) fruit cuticle during development are modulated by changes in the relative amounts of its components. *New Phytol.* 202, 790-802. doi: 10.1111/nph.12727. The average thickness (t) is calculated as: t = w/d

| **daa**  **(day)** | **Weight (w)**  **(µg cm^-2^)** | **Density (d)**  **(g cm^-3^)** | **Average thickness (t)**  **(µm)** |
| --- | --- | --- | --- |
| 15 | 1307 | 1.665 | 7.85 |
| 20 | 1346 | 1.193 | 11.28 |
| 25 | 1456 | 0.965 | 15.09 |
| 30 | 1395 | 0.908 | 15.36 |
| 35 | 1446 | 1.107 | 13.06 |
| 40 | 1367 | 1.110 | 12.32 |
| 45 | 1443 | 1.306 | 11.05 |
| 50 | 1253 | 1.107 | 11.32 |
| 55 | 1332 | 1.221 | 10.91 |

**Supplementary Figure S2.** Correlation between the UV-A blocking capacity of isolated tomato cuticles and their thickness in the low phenolics content developmental stage (15-30 daa).


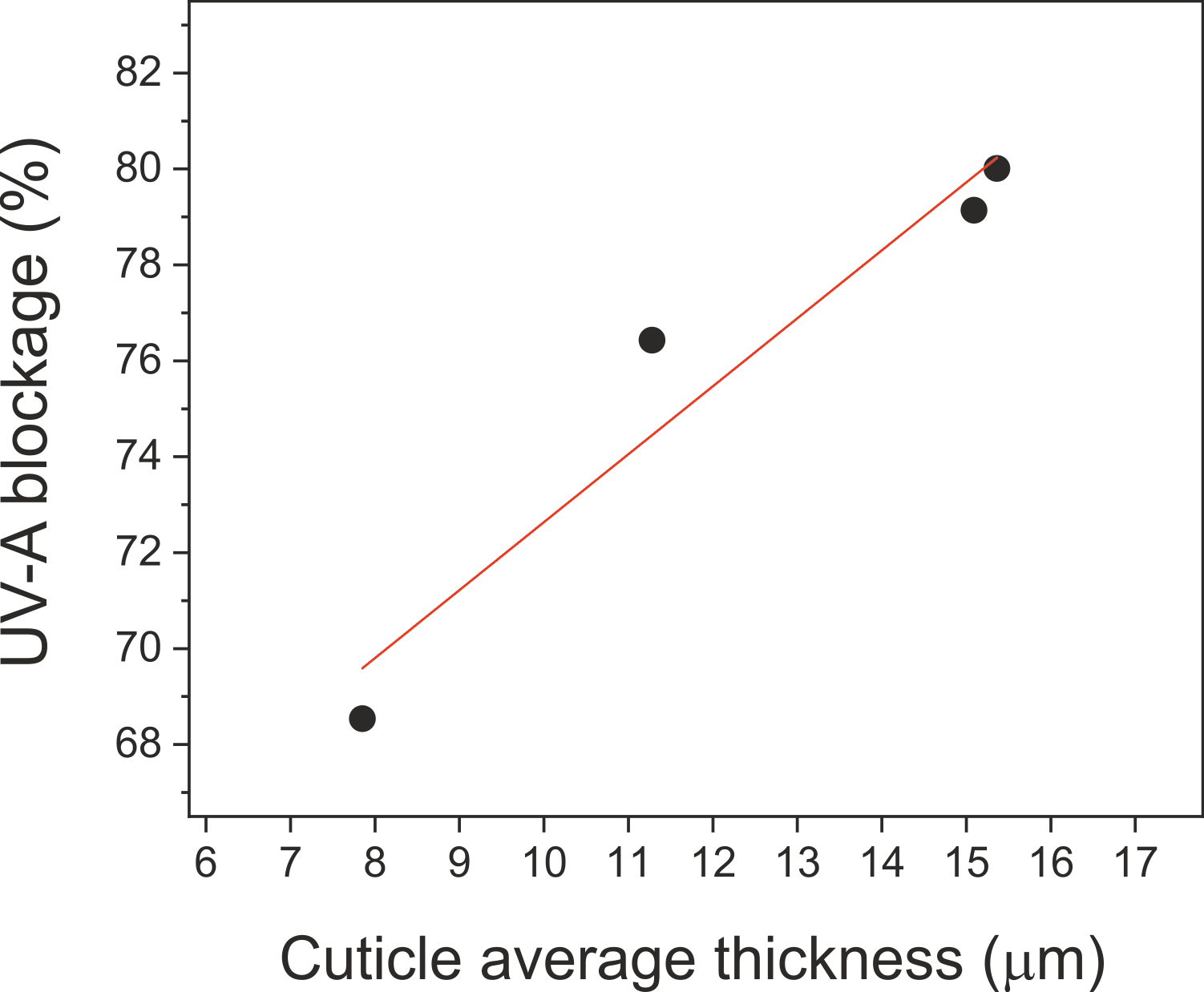

Supplement: Supplementary file 1 [file Data_Sheet_1.docx]
